# Impact of *Pseudomonas aeruginosa* Infection on Patients with Chronic Inflammatory Airway Diseases

**DOI:** 10.3390/jcm9123800

**Published:** 2020-11-24

**Authors:** Marta Garcia-Clemente, David de la Rosa, Luis Máiz, Rosa Girón, Marina Blanco, Casilda Olveira, Rafael Canton, Miguel Angel Martinez-García

**Affiliations:** 1 Pneumology Department, Hospital Universitario Central de Asturias, 33011 Oviedo, Spain; mgclemen@gmail.com; 2 Pneumology Department, Hospital de la Santa Creu i Sant Pau, 08041 Barcelona, Spain; david.rosa23@gmail.com; 3 Servicio de Neumología, Unidad de Fibrosis Quística, Bronquiectasias e Infección Bronquial Crónica, Hospital Ramón y Cajal, 28034 Madrid, Spain; Luis.maiz@lsalud.madrid.org; 4 Pneumology Department, Hospital Univesitario la Princesa, 28006 Madrid, Spain; rmgiron@gmail.com; 5 Servicio de Neumología, Hospital Universitario A Coruña, 15006 A Coruña, Spain; mba@mundo-r.com; 6 Servicio de Neumología, Hospital Regional Universitario de Málaga, Instituto de Investigación Biomédica de Málaga (IBIMA), Universidad de Málaga, 29010 Málaga, Spain; casi1547@separ.es; 7 Servicio de Microbiología, Hospital Universitario Ramón y Cajal and Instituto Ramón y Cajal de Investigación Sanitaria (IRYCIS), 28034 Madrid, Spain; rafael.canton@salud.madrid.org; 8 Pneumology Department, Universitary and Polytechnic La Fe Hospital, 46012 Valencia, Spain; 9 Centro de Investigación en Red de Enfermedades Respiratorias (CIBERES), Instituto de Salud Carlos III, 28034 Madrid, Spain

**Keywords:** *Pseudomonas aeruginosa*, bronchiectasis, cystic fibrosis, COPD, asthma, chronic bronchial infection

## Abstract

*Pseudomonas aeruginosa* (*P. aeruginosa*) is a ubiquitous and opportunistic microorganism and is considered one of the most significant pathogens that produce chronic colonization and infection of the lower respiratory tract, especially in people with chronic inflammatory airway diseases such as asthma, chronic obstructive pulmonary disease (COPD), cystic fibrosis (CF), and bronchiectasis. From a microbiological viewpoint, the presence and persistence of *P. aeruginosa* over time are characterized by adaptation within the host that precludes any rapid, devastating injury to the host. Moreover, this microorganism usually develops antibiotic resistance, which is accelerated in chronic infections especially in those situations where the frequent use of antimicrobials facilitates the selection of “hypermutator *P. aeruginosa* strain”. This phenomenon has been observed in people with bronchiectasis, CF, and the “exacerbator” COPD phenotype. From a clinical point of view, a chronic bronchial infection of *P. aeruginosa* has been related to more severity and poor prognosis in people with CF, bronchiectasis, and probably in COPD, but little is known on the effect of this microorganism infection in people with asthma. The relationship between the impact and treatment of *P. aeruginosa* infection in people with airway diseases emerges as an important future challenge and it is the most important objective of this review.

## 1. Introduction

*Pseudomonas aeruginosa* (*P. aeruginosa*) is a ubiquitous and opportunistic microorganism that represents the paradigm of chronic bacterial infections, where pathogens weaken a host’s defenses and adapt and evolve in order to persist [1]. *P. aeruginosa* finds a favorable ecological niche in people with chronic airway inflammation, thus enabling it to perpetuate itself. The progressive increase in *P. aeruginosa*’s bacterial load has significant deleterious effects and can lead to chronic bronchial infection (CBI), characterized by increased inflammation, both locally and systemically, and an unfavorable clinical evolution.

*P. aeruginosa* is the main cause of CBI in people with cystic fibrosis (CF) and significantly contributes to the low life expectancy [2]. *P. aeruginosa* is, therefore, one of the main targets in therapeutic programs for CF, primarily through the use of inhaled antibiotics [3]. Furthermore, the clinical similarities between CF and non-CF bronchiectasis have led to the discovery by a number of researchers of the frequent presence of *P. aeruginosa* in the latter disease, both during exacerbations and in phases of clinical stability. CBI due to *P. aeruginosa* in people with non-CF bronchiectasis is associated with increased mortality and more frequent hospital admissions and exacerbations, as well as a lower quality of life [4,5]. Recent studies have also confirmed that CBI by *P. aeruginosa* can also occur in people with chronic obstructive pulmonary disease (COPD), particularly in advanced phases of the disease, thereby contributing once again to an unfavorable clinical evolution [6,7,8,9]. In the last few years, various studies have also found bacterial colonization in the lower airways of people with chronic severe asthma. More specifically, *P. aeruginosa* has been isolated in the respiratory secretions of people with long-standing asthma, frequent exacerbations [10], and concomitant bronchiectasis [11].

Accordingly, the presence of *P. aeruginosa* in people with chronic airway inflammation, and more particularly CBI by *P. aeruginosa*, has significant consequences—both clinical and prognostic, but also therapeutic. It is, therefore, important to identify the groups of people who would most benefit from studies focusing on the early detection of *P. aeruginosa*. In this chapter, we review the various implications of CBI by *P. aeruginosa* in people with the most common chronic inflammatory airway diseases: CF, bronchiectasis, COPD, and asthma.

## 2. *Pseudomonas aeruginosa*. Microbiology and Pathogenicity

*P. aeruginosa* is considered one of the most significant of all the pathogens that produce chronic colonization of the lower respiratory tract in people with CF and bronchiectasis, and it is also associated with high morbidity in bronchial exacerbations of people with COPD. From a microbiological viewpoint, the presence and persistence of *P. aeruginosa* over time are characterized by adaptation within the host, followed by a reduced susceptibility to bacterial invasion (albeit with a consolidation of the bacterial presence) that precludes any rapid, devastating injury to the host [12].

In the early stages of colonization, *P. aeruginosa* resembles environmental isolates with typical rough colonies but later they develop a distinctive mucoid morphotype that facilitates the growth of biofilms also observed in non-mucoid strains. This modification has been linked to the persistence of *P. aeruginosa* and the difficulties involved in its eradication, even under antimicrobial treatment with either intravenous or inhaled antibiotics [12,13,14].

Studies of sequential isolates of *P. aeruginosa* in people with CF and bronchiectasis have demonstrated, via various typification techniques, that the specific isolates that enter and colonize the respiratory tract can persist over time [15]. Inter-species comparisons have revealed that adults and children with CF and bronchiectasis are generally colonized by isolates originating from diverse environmental sources [16]. Nevertheless, specific clones—such as, in the case of people with CF, the Liverpool Epidemic Strain (LES, ST146)—have been associated with a poor prognosis and extensive dispersion in people from various CF units in several countries [15,17,18]. Bacterial clones of this kind have several virulence factors that contribute to a deterioration in lung function. These virulence factors have been reviewed in several publications, as summarized in Table 1 [16]. Persistence over time has also been scrutinized, using whole-genome sequencing analysis of sequential isolates recovered from people with CF. This analysis has demonstrated that persistent *P. aeruginosa* adapts itself and modifies the expression of virulence factors, some of which are associated with the typical mucous morpho-type but also with small colony variants [19]. Moreover, this adaptation of *P. aeruginosa* correlates with the emergence of different lineages with distinct evolutionary trajectories that may respond differently to antimicrobial agents, resulting in the appearance of sub-populations or isolates with hetero-resistance in bacterial cultures and susceptibility tests [16,20]. One explanation for this intra-patient diversification is the presence in *P. aeruginosa* of both a core and an accessory genome, the latter originating from mobile elements such as genomic islands that may include phages, transposons, and insertion sequences that could modulate the expression of resistance genes and/or virulence genes [21]. Genome comparisons of PA isolates recovered from different people have also revealed a conserved core genome involving at least 4000 genes in processes such as biofilm formation, antibiotic metabolism, pathogenesis, transport, reduction/oxidation, and secretion systems [22]. Genes associated with virulence and adaptation to the environment are represented in this core genome (Table 1) [16].

Antibiotic resistance is mainly produced in *P. aeruginosa* by mutational events rather than by the acquisition of resistant genes via horizontal transfer [23]. This characteristic is accelerated in chronic infections by the presence of the so-called hyper-mutators, which present alterations in the DNA repair systems and an increased mutation rate (up to 1000 times more than that of wild-type isolates) that allows them to rapidly acquire resistance. This phenomenon has been demonstrated in isolates of CF and bronchiectasis, where the frequent use of antimicrobials facilitates the selection of hyper-mutators [18]. The presence of a hypermutator phenotype increases the genetic diversity of each subsequent bacterial generation, which in turn increases the probability of producing sub-strains carrying resistance to antimicrobials. The identification of hyper-mutators in a clinical microbiology laboratory has been recommended, as their presence is associated with reduced lung function [24,25].

## 3. *Pseudomonas aeruginosa* in Cystic Fibrosis

### 3.1. Prevalence and Risk Factors for Infection

*P. aeruginosa* is the most prevalent potentially pathogenic microorganism (PPM) in CF. The rate of infection increases with age, but the percentage of people with CF with a positive sputum culture for *P. aeruginosa* has dropped in recent years. Although the risk factors for PA infection are not entirely known, those that have been most widely recognized to date are chronic bronchial infection by Staphylococcus aureus [26] and its treatment [27,28], female gender [26], and contact with other people chronically infected by *P. aeruginosa* [29].

### 3.2. Detection of Infection

It is difficult to identify primary infection by *P. aeruginosa* (understood as the first isolation of PA in a respiratory sample culture) in the lower respiratory tract of people with CF due to limitations in culturing techniques (the culture of spontaneous or induced sputum, oropharyngeal and endolaryngeal aspiration, and bronchoalveolar lavage) and patients’ failure on occasions to expectorate (particularly small children).

Although no single technique can be considered a gold standard, as each has its advantages and disadvantages, the most widely used methods at present are that of spontaneous sputum in children aged over 6 years and oropharyngeal aspiration in younger children. However, aspiration has less sensitivity and specificity for predicting infection of the lower respiratory tract by *P. aeruginosa*, while broncheoalveolar lavage is an invasive technique which, moreover, can present false negatives as different lobes of the lung can be affected in different ways [30]. In the case of a child with CF, a negative throat swab has a negative predictive value for *P. aeruginosa* in the lower respiratory tract of 95%, making it clinically quite useful [31].

### 3.3. Adaptation in the Airways

People with CF are usually infected initially by single isolation of *P. aeruginosa*. CBI ensues after a subsequent period of intermittent isolations in the lower respiratory tract. This transition from intermittent to chronic infection intensifies both local and systemic inflammation, thereby damaging the lung. Accordingly, immediate efforts must be made to eradicate *P. aeruginosa* via antibiotic treatment to prevent it from acquiring the mucous phenotype and growing the biofilms that lead to a CBI, at which point eradication becomes a great deal more difficult [32].

### 3.4. Impact of Infection

Primary *P. aeruginosa* infection does not seem to precipitate any decline in lung function, so in the early years, its clinical manifestations can be subtle, ranging from a slight decline in lung function [33,34,35] to the clinical and radiological deterioration [33,34]. It is striking, however, that the morbidity associated with primary infection cannot always be reversed, even by early, aggressive anti-pseudomonal treatment [36]. According to some authors, it is difficult to distinguish between *P. aeruginosa* infection as a marker of underlying disease risk versus a causal agent of disease. In fact, although eradication of first CF *P. aeruginosa* infection is the standard of care in Europe and North America, there is scant evidence that this provides a clinical benefit. Mayer-Hamblett et al., in a 5-year follow-up study of over 200 people with CF who underwent *P. aeruginosa* eradication treatment, observed and concluded that although there were differences in antimicrobial usage between individuals who were converted to *P. aeruginosa* culture negativity versus those who were not, there was no association between eradication status and clinical outcomes including rate of exacerbation and lung function decline over 5 years following treatment [37]. The truth probably lies in the middle and people destined to have poor outcomes are probably at greater risk of *P. aeruginosa* infection because of their underlying biology, and infection probably further drives their disease.

Alterations in the CF transmembrane conductance regulator (CFTR) protein trigger changes in the rheology of the mucus and the mucociliary clearance, as well as an exaggerated inflammatory reaction primarily confined to the airway. The bronchial tree is obstructed by secretions that impede the elimination of PPM and thus facilitate chronic infection. The progress of the lung disease is determined by changes in the microbiome despite its immune capacity [38]. A healthy lung microbiome mainly comprises Bacteroidetes and Firmicutes, which migrate toward the lung from the oropharynx through the aspiration of saliva [39], but in CF it can vary from patient to another in terms of structure, composition, and diversity [40]. The core microbiota consists of five genera: Streptococcus, Prevotella, Rothia, Veillonella, and Actinomyces. In the initial phases of CF, there is a greater diversity of bacteria, and the main pathogens associated with the disease are Staphylococcus aureus and Haemophilus influenzae. Other pathogens that appear as the disease progresses, such as Pseudomonas, Burkholderia, Stenotrophomonas, and Achromobacter, are less prevalent than the core genera, but they have a strong tendency to dominate the bacterial community once they are present [41]. *P. aeruginosa* displays a significant resistance to both innate immune effectors and antibiotics. This is partly because it expresses significant virulence factors (e.g., antioxidants and exopolysaccharides, biofilm growth) and acquires spontaneous mutations with a selection of variants more phenotypically suited to long-term airway colonization [42].

The factors that influence the microbiome composition of CF include the patient’s age, treatment with antibiotics, and protein therapy. Generally speaking, the lung’s microbial diversity decreases with age in CF and chronic infection is triggered by one or two pathogens, most notably *P. aeruginosa* [39], while this diversity, and the lung function itself, is better in children aged under 10 years [43]. Reduced diversity in the microbiota of the lung and the predominance of *P. aeruginosa* are independently associated with greater inflammation, more frequent exacerbations, a rapid decline in lung function, and progression to severe forms of CF that require a transplant, as well as a higher mortality rate [38,42,44,45]. Table 2 summarizes the studies that have analyzed the various consequences of infection by *P. aeruginosa* in people with CF.

It has been reported that a high percentage of people with CF aged over 18 years present chronic infection by *P. aeruginosa* [37]. According to the data in the Cystic Fibrosis Foundation Patient Registry [46] in the United States, *P. aeruginosa* was isolated in 55% of adult people with CF, while in the European registry (https://www.ecfs.eu/ecfspr) 30% of people had an infection due to PA (13% in children and 45% in adults). CF units in Spain have reported CBI due to PA in 46% of people with CF (29% in children and 63% in adults), and this is associated with poorer lung function [47]. The isolations of *P. aeruginosa* in Spain are extremely diverse and genetically unrelated, with numerous combinations of virulence factors and high rates of resistance to antimicrobials (except for colistin) [48]. Moreover, the earlier the initial PA infection, the poorer the prognosis and the faster the evolution of the disease [49,50].

CBI due to *P. aeruginosa* is the most important cause of morbidity and mortality in people with CF [43,75,76,77,78,79]. It is associated with more respiratory symptoms [27,80], a greater number of exacerbations [49,50,61,62,81], increased inflammation (with higher concentrations of neutrophil elastase in the sputum), higher levels of serum C reactive protein (CRP) [54], and reduced monocyte response to various stimuli [51]. The lung function is also poorer [69,73,76,77], with more obstructive functional patterns [52] and greater deterioration [69,79,82]. It has been observed that people with CF with no PA infection, or a successfully eradicated infection, present an annual forced expiratory volume in 1 s (FEV1) loss (as a percentage of predicted value) of 1.65%, while a drop of 4.74% has been found in those with chronic infection [71]. There have also been reports of increased structural damage in CT [50,59,68], with a greater progression of air entrapment and bronchiectasis [60], and of a close correlation between lower CT scores and the acquisition of *P. aeruginosa* [27,50,59,68,80]. There are some discrepancies in the results published on the impact of PA infection on the quality of life [58,83,84,85], as some authors found no differences between infected and uninfected people with CF [58,80,83], while others did find them when people were infected by specific strains, such as the Liverpool Epidemic Strain [64]. Furthermore, the appearance of phenotypic variants, such as mucoid forms or small colonies that are rough or show deficient motility, accelerate a patient’s decline still further [53,64]. The change from a non-mucoid to a mucoid morpho-type is associated with a lower weight percentile, a greater number of exacerbations and hospitalizations, a greater and faster lung function decline, a greater progression of structural damage, a worsened quality of life, and a higher mortality rate, although theses analysis are confounded by the duration of chronic infection [49,50,72,78,86,87,88].

Finally, and most importantly, many studies have shown that the progression to severe forms of CF and then death is quicker in people with CBI by *P. aeruginosa*, compared to those that remain uninfected [70,74,89,90].

As mentioned in the previous section, the isolation of *P. aeruginosa* in people with CF results in greater inflammation, as manifested by elevated levels of elastase in the neutrophils in the sputum and serum PCR, a higher number of exacerbations, poorer lung function, an increase in respiratory symptoms, a worsened quality of life, greater structural damage, and a higher mortality rate [55,56,57,63,65,66,67].

Accordingly, eradication has become the primary objective. It has been shown that 2–3 weeks of early treatment with inhaled antibiotics [91,92,93], usually in association with systemic antibiotics active against *P. aeruginosa* [13,94,95], achieves high rates of eradication and delays CBI.

The first studies with colistin [96,97] demonstrated a reduction in the rate of chronic colonization with respect to placebo and fewer hospital admissions than in historic controls and small series with no control group [98]. Numerous studies have also shown the eradicative effects of tobramycin, usually administered for inhalation in solution (TIS), in on-off cycles of 28 days [93,99]. The efficacy of colistin and tobramycin has proved similar with respect to their eradication rates and effects on lung function [100,101]. Benefits have also been observed from treatment with aztreonam in primary infection by *P. aeruginosa* [102] in children aged from 3 months to 18 years. A review undertaken by Cochrane provides an analysis of the different strategies used [103].

In conclusion, an initial infection by *P. aeruginosa* is often eradicated in both young people (68–93%) and adults (79%), using a variety of antipseudomonal regimens, with a mean time before any reappearance of infection of 8 to 18 months [93,100,101,104]. There is an increased probability of eradication if treatment is administered before the development of CBI [105,106].

When bacteria cannot be eradicated, attempts can be made to reduce the bacterial load [107] and thus prevent damage caused by the inflammatory response. The first studies on inhaled colistin and tobramycin have shown improvements in the symptoms of people with CBI and less deterioration in lung function [108,109,110]. The introduction of TIS in on-off 28-day cycles has reduced the density of *P. aeruginosa* in the sputum and improved the lung function [111,112,113], as has the dry powder formulation of tobramycin (TIP) [114]. Comparative studies of colistin and inhaled tobramycin [115,116] found improved lung function and symptoms, drops in PA levels in the sputum, and fewer exacerbations, although they also showed some discrepancies (probably attributable to methodological factors). Inhaled aztreonam has also proved efficacious in the treatment of CBI by *P. aeruginosa*, in both mild and moderate–severe cases of CF [117,118,119,120], with improvements in the FEV1 and reductions in the number of PA colonies. In the AIR-CF1 study, the effects on the quality of life were greater in children aged under 18 years than in older ones. One study that compared aztreonam with TIS30 showed statistically significant differences in favor of aztreonam, with improvements in the FEV1 and the quality of life (SR-CFQ-R), longer periods free from exacerbation, a reduced need for antibiotic treatment, and weight gains. A review undertaken by Cochrane [121] found benefits that included reductions in the bacterial load in the airways and in the numbers of exacerbations and hospital admissions, as well as improved lung function, lower mortality rates, and better quality of life.

Other inhaled antibiotics that have appeared more recently include levofloxacin (LF) [122] and liposomal amikacin [123]. Nebulized LF significantly reduces the levels of both *P. aeruginosa* in sputum and the administration of antibiotics, as well as improving the FEV1 and showing a tendency to alleviate symptoms after 28 days of treatment. One phase 3 study evaluating the efficacy and safety of LF against TIS found them to be comparable as regards both FEV1 and the time-lapse prior to the first exacerbation, and it concluded that LF is as safe and efficacious as TIS and, therefore, provides an alternative for people with CF and CBI due to *P. aeruginosa* [124].

Liposomal amikacin has been shown to improve lung function, reduce the density of *P. aeruginosa* in sputum, alleviate respiratory symptoms, and enhance the quality of life [123]. It was comparable to TIS with respect to the FEV1 at the end of three cycles. Furthermore, the most recent Cochrane review [125] observed improvements after the administration of liposomal amikacin, primarily in the number of exacerbations and the lung function.

In conclusion, inhaled antibiotics in their various formulations have demonstrated significant benefits to people with CF through the alleviation of symptoms, reduction of the bacterial load and the number of exacerbations, prevention of progressive deterioration of lung function, and improved quality of life.

## 4. *Pseudomonas aeruginosa* in Non-Cystic Fibrosis Bronchiectasis

### 4.1. Primary Infection. Impact on the Clinical Picture and Prognosis

*P. aeruginosa* is the potentially pathogenic microorganism (PPM) that has been most widely studied in bronchiectasis but there still remains much to learn about its natural history and the epidemiology of bronchial infection in this disease [126]. Many of the notions about the behavior of *P. aeruginosa* in the airways of people with bronchiectasis have been extrapolated from CF, but there are certain differences between the two diseases. For example, while bronchial infections in adults with CF tend to be caused by the same strain of *P. aeruginosa*, this is not true of non-CF bronchiectasis [127,128] nor is it common to find crossed infection by *P. aeruginosa* in people with bronchiectasis, but this is the case with CF [127,129,130].

In both diseases, however, it is difficult to ascertain the true impact of primary infection by *P. aeruginosa* in people with bronchiectasis, as most studies have overlooked this factor to concentrate on CBI.

A number of different individual variables have been associated with a poor prognosis for people with bronchiectasis. These include age, dyspnea, the quantity and purulence of sputum, a lower body mass index (BMI), airflow obstruction and the number of lobes affected, as well as exacerbations, systemic inflammation, comorbidities, and colonization/CBI by potentially pathogenic microorganisms, especially *P. aeruginosa* [131].

The presence of *P. aeruginosa* in the airways of these people has been linked to a more marked decline in lung function [132,133], although some authors have suggested that PA colonization may not be directly associated with a poorer lung function [134,135,136]. Other factors that have been associated with this phenomenon include reduced quality of life [137], increased morbidity and mortality [136,137,138,139,140], greater local and systemic inflammation [141], higher medical costs [142], and a higher mortality rate [138] (Table 3).

Some research groups have recently investigated the predictive power of *P. aeruginosa* and other variables in order to create the multidimensional severity scores FACED (the acronym for FEV1, Age, PA Colonization, radiological Extension of bronchiectasis and Dyspnea) and the Bronchiectasis Severity Index (BSI). Both these scores have presented an excellent capacity to predict all-cause mortality, new exacerbations, and long-term respiratory mortality. This is generally much more useful than the information that can be gleaned from a separate evaluation of each variable [131,144]. Both scores show that colonization by *P. aeruginosa* is one of the variables that predicts a poorer prognosis. This could be due to various factors, particularly the bacteria’s production of toxic substances such as hydrogen cyanide [150] and hydrogen peroxide [151], intense bronchial inflammation [152], and an increase in the daily production of sputum.

Since CBI by *P. aeruginosa* is associated with a poorer prognosis for people with bronchiectasis, the Spanish and international guidelines recommend, on the basis of the good results achieved in eradicating *P. aeruginosa* in CF, that these people should be administered an aggressive eradicative treatment as soon as PA appears in their respiratory samples [153,154]. This treatment needs to be prompt, forceful, and prolonged.

### 4.2. Chronic Bronchial Infection. Clinical Impact, Lung Function, Quality of Life, Mortality and Other Factors

The airway of a patient with bronchiectasis gives rise to an ecological niche ideally suited to CBI, as a result of alterations in the mucociliary clearance system and deregulation of the immune system. The most frequently isolated PPM are Haemophilus influenzae (20–40%), *P. aeruginosa* (10–30%) and, to a lesser extent, other gram-negative (Moraxella catarrhalis, Escherichia spp and Klebsiella spp) and gram-positive bacteria (Streptococcus pneumoniae and Staphylococcus aureus) [153,154,155]. Of all these, *P. aeruginosa* is the most significant due to its microbiological characteristics (virulence factors, formation of biofilms, and hypermutability) (Table 1) and its clinical consequences (Table 3). CBI by *P. aeruginosa,* therefore, provides the basis for a specific clinical phenotype and constitutes an item that can be evaluated on the main multidimensional prognostic scores. Its prevalence varies from series to series and country to country, and it has been suggested that these discrepancies are due to environmental factors [156].

#### 4.2.1. Clinical Impact

Various studies dating back more than 30 years reported a negative clinical impact of *P. aeruginosa* on people with bronchiectasis, although it proved difficult to demonstrate this reliably, owing to the particular characteristics of the studies and the heterogeneity of the variables involved in the prognosis of bronchiectasis [134,135,143,146,157]. More recently, several authors have tried to group bronchiectasis into clusters or phenotypes [147,158,159,160,161,162,163,164], of these, the most solid are those of the patient with CBI by *P. aeruginosa* [160] and the frequent exacerbator [161,162]. Aliberti et al. described four clinical phenotypes in 1145 people with bronchiectasis in various European countries, with a follow-up of 3 years, and the cluster with *P. aeruginosa* was associated with the most symptoms, the most cough (95%) and daily expectoration (93%), the presence of hemoptysis (24%), the most dyspnea on the Medical Research Council (MRC), and the greatest number of exacerbations and hospitalizations in the previous year (61%) [147].

The exhaustive systematic review was undertaken by Finch et al. in 2015 [140], which analyzed 21 observational studies that included 3683 people with bronchiectasis; the rates of hospitalization for these people ranged from 41% in the first year and 75% in the fourth, while in those without bronchiectasis these rates were 15% and 28.5%, respectively. The odds ratio of hospitalization for CBI by *P. aeruginosa* was 6.57 (95% CI, 3.19–13.51).

Furthermore, there is evidence that exacerbations have a negative impact on the natural history of bronchiectasis, and the exacerbator phenotype represents a significant cluster. The number and, above all, the severity of exacerbations has been associated with greater disease severity, poorer quality of life, greater risk of CBI, a rapid decline in lung function, greater systemic inflammation, higher costs, and more premature deaths; all these factors make it a top priority in the therapeutic and preventive strategies for bronchiectasis [161,162]. The mean of annual exacerbations is 1.29 ± 0.9 in the absence of CBI, but this figure rises to 2.04 ± 1.4 in the presence of CBI and to 2.85 ± 1.5 when CBI is caused by *P. aeruginosa*, and it is even higher in cases of S. aureus resistant to methicillin [158]. However, there is a small subgroup of people with CBI by *P. aeruginosa* with no pulmonary exacerbations and less morbidity-mortality, but the factors that influence this particular clinical circumstance have yet to be elucidated [139,147,161].

#### 4.2.2. Impact on Lung Function

Of all the parameters of lung function, FEV1 is the most widely accepted as a prognostic marker of airflow obstruction. In the case of bronchiectasis, an annual FEV1 loss between 39 and 55 mL/year [1.43% to 2.35%] has been reported [5,6,9,18,20]. A study by Olveira et al. of the Spanish historical registry for bronchiectasis [165] found that the risk factors associated with worsened lung function were female gender, age, a lower BMI, and the presence of CBI. In a group of 76 people with well-characterized bronchiectasis and 2 years of follow-up, Martínez-Garcia et al. described CBI by *P. aeruginosa*, frequent severe exacerbations, and increased systemic inflammation as independent factors in lung function decline [132]. People with CBI by *P. aeruginosa* presented a greater drop in annual FEV1 than uncolonized people (123.3 mL/year (−5.53%) vs. 30.8 mL/year (−1.38%), respectively) [132]. Similarly, most studies have observed lower FEV1 values in people with *P. aeruginosa* (ranging from −1.4% to −29%) than in their remaining people [140]. However, Davies et al. concluded in their study of 163 people with bronchiectasis (82 with intermittent isolates and only 14 with CBI) that *P. aeruginosa* was associated only with poorer lung function and not with a deterioration in FEV1 [136]. In contrast, a recent study based on data from 849 people in the computerized Spanish registry of people with bronchiectasis (RIBRON) described an annual FEV1 loss of −31.6 mL and found that factors such as age, CBI by *P. aeruginosa*, severe exacerbations, and higher values of FEV1 affected this deterioration [133].

#### 4.2.3. Impact on the Quality of Life

Quality of life is a very important parameter in the evaluation of the overall impact of bronchiectasis [85,166] and, accordingly, it has been one of the main outcomes in many clinical studies of the disease. People with bronchiectasis tend to present a poorer quality of life than the general population [140,143]. Various factors have been linked to this finding: age, chronic colonization by *P. aeruginosa*, the degree of dyspnea, poorer lung function, the number of exacerbations, bronchial hyperreactivity, greater structural damage, daily bronchorrhea, respiratory insufficiency, and symptoms of anxiety and depression [85,166,167]. Finch’s review concluded that, according to the Saint George’s Respiratory Questionnaire (SGRQ), people with bronchiectasis with CBI by *P. aeruginosa* had a quality of life 18.2 points higher than those without [140]. A higher proportion of symptoms of anxiety and depression has been observed in people with bronchiectasis than in the general population, and this altered psychological morbidity has repercussions on their quality of life [168]. Similarly, people with CBI by *P. aeruginosa* have presented higher scores for anxiety [169].

#### 4.2.4. Impact on Mortality

A multi-variant study by Loebinger et al. was one of the first to evaluate factors that influence the survival of people with bronchiectasis. It included 91 adults with a follow-up of 13 years and found that age, quality of life, CBI by *P. aeruginosa,* and some functional parameters were factors independently associated with mortality [138]. However, in another subsequent multi-variant analysis of mortality in 245 adults with bronchiectasis followed up for 5 years, Goeminne et al. found that the only significant factors were COPD and the number of lobes affected [145].

The poor prognosis associated with CBI by *P. aeruginosa* was confirmed in Finch’s review [140], where the adults with bronchiectasis with CBI by PA presented a three times greater risk of mortality than the other people. The mortality rate in the former was 7.7% in the first year, 13.6% in the second, and 30–50% at 5 years, whereas these figures were 0%, 7%, and 9–15% in the adults without *P. aeruginosa*. The overall odds ratio for CBI by *P. aeruginosa* was 2.95 (95% CI, 1.98–4.40). The nature of this type of study does, however, preclude any control of all the confounding factors involved, due to the heterogeneity of the design of the studies that it covered.

Araujo et al. [149] analyzed the long-term implications of *P. aeruginosa* in 2596 bronchiectasis adults (15% with CBI by PA) in 10 centers in Europe and Israel, with 5 years of follow-up. These authors showed that CBI by *P. aeruginosa* is not independently associated with mortality unless it is also associated with two or more exacerbations.

The importance of CBI by *P. aeruginosa* for prognosis has been reflected in the three multidimensional scores that have been validated to assess the severity of bronchiectasis and predict both exacerbations and mortality: the FACED [131], E-FACED [148], and BSI [144]. All three include CBI by *P. aeruginosa* as a prognostic item, with a score ranging from 1 point in FACED (0–7) and E-FACED (0–9) to 3 points in BSI (0–26).

#### 4.2.5. Other Factors

CBI by *P. aeruginosa* has also been associated with radiological progression. One study by Park that included 155 people with bronchiectasis with a follow-up of over 5 years observed radiological deterioration, as quantified by the Bhalla score for computerized axial tomography. The multi-variant analysis showed the factors involved to be a lower BMI and isolations of *P. aeruginosa* [170].

People with bronchiectasis engender high healthcare costs, which, according to a Spanish multi-center study by De la Rosa [142], are independently associated with age, the percentage of FEV1, CBI by *P. aeruginosa,* and the number of hospitalizations. Patients with CBI by *P. aeruginosa* gave rise to mean annual costs roughly as high as those derived from those with severe bronchiectasis (8654.40 €) [142].

Increased local and systemic inflammation is related to the bacterial load in people with bronchiectasis, but it is independent of this load in those with *P. aeruginosa* [141].

In conclusion, CBI by *P. aeruginosa* has a significant impact on people with bronchiectasis, owing to its prevalence and clinical consequences, as well as its microbiological characteristics. It features as an item on multidimensional prognostic scores and constitutes a phenotype in its own right. More studies based on data from patient registries and biological markers are required, however, to clarify the involvement of *P. aeruginosa* in the natural history of bronchiectasis.

### 4.3. Responses to the Treatment of Primary Chronic Bronchial Infection in People with Bronchiectasis

PA isolations have a negative impact on a non-CF bronchiectasis patient [132,135,171] but long-term antibiotic treatment has a positive impact, as it reduces airway inflammation and lowers the risk of exacerbations [141]. Therefore, as with people with CF, efforts must be made to administer an early eradicative treatment, although studies of such treatments in non-CF bronchiectasis are scarce and very heterogeneous [172,173,174,175,176,177]. Two studies of tobramycin [172,177] with very small numbers of adults showed eradication rates of 35% at 6 weeks and 54.5% after 15 months. In another study, this figure was 22.2% at 12 weeks [174]. The few studies undertaken with inhaled colistin included no more than 30 patients each [173,175,176], and only one [173] had an eradicative program involving systemic antibiotics (its eradication rate was 43% at 14 months). One 12-month prospective cohort study with a protocol for the eradication of *P. aeruginosa* comprising the administration of systemic antibiotics followed by inhaled colistin (Promixin 1 MIU twice daily administered via I-neb^®^; Philips Respironics, Chichester, UK) [178] reported eradication rates of 61.2%, 50.7%, 43.3%, and 40.3% after 3, 6, 9, and 12 months of treatment, respectively.

Despite the limited amount of data available, the main clinical guidelines recommend the use of inhaled antibiotics for treating an initial infection by *P. aeruginosa* [12,13].

As regards CBI by *P. aeruginosa*, inhaled tobramycin has achieved a temporary eradication in some cases [174,179,180,181] and a reduction of the bacterial load in most cases [174,177,179,180], as well as a drop in the number and duration of hospitalizations [177,181], a degree of clinical improvement [179,180], and better quality of life [174]. A controlled randomized study with gentamicin [182] found reductions in inflammatory markers, the bacterial load, and the number of exacerbations, along with an eradication rate of 30.8%.

The first published study on colistin, in 18 people (14 with CBI by *P. aeruginosa*), reported a slower decline in lung function and improvements in the quality of life, without any reduction in the rate of hospitalizations. Another small, uncontrolled retrospective study [175] observed reductions in the rates of exacerbations and hospitalizations, the number of positive sputum cultures, and the volume of sputum, but lung function was unaffected. However, another controlled, open, prospective study [183] did not find any differences after one year of treatment in the number or duration of hospitalizations, or in the FEV1 or dyspnea.

The greatest body of evidence, however, has been provided by another study that included 144 adults (73 treated with colistin and 71 with placebo) [184]. It observed a reduction in the density of *P. aeruginosa* and improved quality of life compared to placebo in those who complied with at least 80% of the treatment. It also found a long time period prior to the next exacerbation. Similarly, another uncontrolled study [178] showed a significant reduction in both exacerbations and hospital admissions.

The combination of colistin and tobramycin has been associated with shorter hospital stays and a need for antibiotic treatments, as opposed to monotherapy [185]. Studies of aztreonam [186] have found a significant reduction in the bacterial load, but only AIR-BX2 achieved any significant improvement in the quality of life, as measured by the quality of life (QOL)-B questionnaire. A recent meta-analysis [187] of all the randomized controlled studies of inhaled ciprofloxacin (two phase 2 studies and four phase 3 studies), covering 1685 adults (1094 with inhaled ciprofloxacin and 591 with placebo), found a clinical benefit in terms of fewer exacerbations in people with non-CF bronchiectasis. The latest meta-analysis of inhaled antibiotics in non-CF bronchiectasis [188] concluded that these drugs’ greatest benefits are to be found in their reduction of the bacterial load [180,182,184,189,190,191] and both the time leading up to the next exacerbation and the frequency of exacerbations [182,189,192,193]. There were greater variations between the results of the different studies with respect to the eradication rates [141,177,180,182,184,185,186,190,191,192,193], effect on the lung function [141,177,180,181,182,184,190,191,192,193], and quality of life, as measured by the SGRQ [177,181,184,190,191,192,193], QoL-B [186,192,193] and Leicester Cough Questionnaire (LCQ) [182]. These discrepancies can be explained by the different etiologies of bronchiectasis, the varying proportions of PPM other than *P. aeruginosa*, the geographical locations in countries with distinct cultures and health systems, the varying times and proportions of people with macrolides, the absence of reliable methods for measuring adherence to treatment, and differences in the definitions of exacerbation, in the numbers of previous exacerbations, and in the antibiotic schedules and nebulization systems.

Although the clinical benefits of inhaled antibiotics have not been as consistent in bronchiectasis as they have been in CF, the general consensus is that the negative repercussions and high prevalence of *P. aeruginosa* suggest that there are reasonable grounds for their use, and accordingly this is recommended in the main guidelines [153,155].

## 5. *Pseudomonas aeruginosa* in COPD

Cross-sectional studies have shown that *P. aeruginosa* accounts for 4–15% of all the PPM capable of inducing CBI in COPD [6,194]. Multiple risk factors have been identified for PA infection in COPD: previous isolation of *P. aeruginosa*, multiple courses of systemic antibiotics or steroids, more advanced disease, bronchiectasis, current smoking habit, and a previous stay in an intensive care unit [6,194,195,196,197,198]. However, the relationship between the isolation of *P. aeruginosa* and poor outcomes in people with COPD is more controversial. Jacobs et al. prospectively studied 181 people with COPD, 40% of whom had PA isolation. Both the first isolation and multiple isolations of *P. aeruginosa* were linked to higher mortality [9]. Conversely, Boutou et al. [199] concluded that single isolation of *P. aeruginosa* is not associated with higher mortality in people with COPD. As regards exacerbations, Rosell et al. [200], after pooling the results of six studies that obtained microbiological samples via a protected specimen brush, observed that *P. aeruginosa* was associated with a greater number and severity of exacerbations, regardless of the bacterial load. However, Murphy et al. [201] concluded, in a 10-year prospective study, that only the acquisition of a new strain of *P. aeruginosa* (and not all positive cultures) was associated with an increased incidence of exacerbations. More recently, Eklöf et al. [8] carried out an epidemiological study in 22,053 people with COPD, 4.2% of whom had at least one positive culture for *P. aeruginosa*, and they found that *P. aeruginosa* strongly and independently predicted an increased risk of hospitalization and all-cause death. However, some of the analyses of the exacerbations and mortality related to *P. aeruginosa* obtained their microbiological samples during an exacerbation period [202,203,204,205,206,207,208].

One of the most interesting debates at the moment is whether *P. aeruginosa* is a marker of disease severity or a cause of exacerbations and rapid deterioration in people with COPD. Although there is still no clear answer to this question, Martinez-Solano et al. [206] have provided some evidence supporting the latter hypothesis after observing patterns of PA infection and development in COPD resembling those found in CF. Yet again, however, the lack of agreement on this topic is illustrated by another study, by Rakhimova et al. [209], which showed that the *P. aeruginosa* found in COPD has a frequent turnover of different clones distinct from that found in CF (which are usually a chronic carrier of the same PA), with the mucoid form being most frequent.

A better understanding of the influence of PA infection on COPD morbidity and mortality in outpatients, and the experience gained from treating both bronchiectasis and CF, would help us to implement specific therapies and new procedures for the prevention, diagnosis, and treatment of PA infection in people with COPD.

## 6. *Pseudomonas aeruginosa* and Asthma

Many studies have been published on CBI by *P. aeruginosa* in bronchiectasis and people with COPD but only a few have evaluated the isolation of bacterial pathogens in the respiratory secretions of people with asthma. People with severe asthma can present both bronchiectasis and CBI by various pathogens, but they are often not subjected to a microbiological study, thereby complicating the management of their disease and hindering a successful therapy [210,211]. In such people, the presence of uncontrolled bronchial inflammation can lead to increased production of respiratory secretions, with a consequent formation of mucous blockages, leading to local damage to the bronchial mucus and *P. aeruginosa* the development of bronchiectasis. People with coexisting bronchiectasis and asthma tend to be older, with a longer duration of asthma, greater airway inflammation and functional decline, more frequent exacerbations and, therefore, greater disease severity [211,212,213,214,215,216,217,218,219]. Studies that have analyzed the cultures of respiratory secretions of people with asthma have found that the presence of bacteria in the sputum is associated with a longer duration of the disease, poorer lung function (FEV1), and more neutrophils and a higher concentration of Il-8 in the sputum [219,220]. 

The respiratory microbiome has been shown to play an important role in the response of people with asthma response to treatment. A study by Durack et al. observed that people who responded badly to inhaled corticoids had greater dysbiosis in the respiratory microbiome, while those who responded favorably had a respiratory microbiome similar to that of healthy controls [221].

Overall, few studies have evaluated bacterial isolations in the sputum of people with asthma. One study undertaken by Sánchez-Muñoz et al. [222] analyzed the presence of bronchiectasis in people with asthma who had been discharged after an exacerbation and found a prevalence of 3.02%. Eight percent of people with concomitant bronchiectasis also presented CBI; this figure was much higher than that in the people with asthma without bronchiectasis. CBI by *P. aeruginosa* also proved to be a predictor of mortality (OR: 1.67 1.35–2.06). Dimakou et al. [216] studied 40 adults with asthma and collected sputum for bacterial cultures from all of them. In nine cases (22.5%), one or more pathogens were isolated in the sputum, the most common being *P. aeruginosa* and H. influenzae. All the people with pathogens in their sputum had bronchiectasis, and this group was also taking more antibiotics than the group without bronchiectasis. Mao et al. [223] investigated the influence of asthma on exacerbations of bronchiectasis and found isolations of *P. aeruginosa* in the respiratory secretions of 19.7% of the 214 adults under study (all with co-existing bronchiectasis and asthma). Zhang et al. [224] studied the isolation of bacterial pathogens in 56 adults with severe asthma and obtained positive bacteria cultures in 29 of them (52%), with H. influenzae the most frequently identified, followed by *P. aeruginosa* and S. aureus. The presence of positive cultures was associated with a longer duration of asthma and exacerbations in the previous year. 

Padilla et al. [218] studied 398 adults with asthma (60% with severe asthma) and found bronchiectasis to be present in 28.4% of them, as well as positive sputum cultures in 29.7% of them with both diseases and in 21.1% of those with asthma alone. The coexistence of bronchiectasis and asthma was associated with the presence of chronic bronchial expectoration and purulent sputum.

The isolation of bacteria in the respiratory secretions of people with asthma has also been associated with the presence of allergic bronchopulmonary aspergillosis (ABPA) [225]. A study by Ishiguro et al. [226] observed 42 people with ABPA unrelated to CF and found CBI due to S. aureus in 35.7% of them and CBI due to *P. aeruginosa* in 19%. The presence of bronchiectasis and the use of high doses of corticoids to treat ABPA (administered via inhalation or systemically) were the factors that predisposed these people to respiratory infections. Another study by Tomomatsu et al. [225] evaluated 25 adults with asthma and ABPA, of whom 6 (24%) presented CBI by *P. aeruginosa* and a further 6 (24%) CBI by atypical microbacteria. They all received treatment with moderate or high doses of inhaled corticoids, and two-thirds of them also received systemic corticoids. However, no other reports of such a high prevalence of concomitant infection in people with asthma and ABPA have been published outside Japan. This could be because the people were more elderly and the appearance of ABPA later in life made them more vulnerable to chronic respiratory infections.

In short, few studies to date have investigated the isolations of bacterial pathogens in the sputum of people with asthma. There have been increasing reports, however, of bronchiectasis in these adults, mainly in elderly people with long-term asthma and poorer control of the disease, as well as a weaker functional state. Analysis of the presence of bacterial pathogens in these adults’ sputum could be a worthwhile field of exploration for improving the management and evolution of their disease.

## 7. Future Challenges

The progressive increase in studies on the relationship between *P. aeruginosa* and chronic respiratory diseases (particularly COPD and non-CF bronchiectasis) has answered some questions, but these publications have also opened up new issues that need to be tackled in the coming years [227]. Large-scale, long-term studies must be conducted if we are to draw any firm conclusions about the clinical evolution of these diseases and strategies for diagnosing and managing them. We still do not know whether CBI by *P. aeruginosa* is merely a marker of the severity of non-CF bronchiectasis and COPD, or whether it causes an increase in the number of exacerbations. If the latter is the case, then early treatment could prevent these exacerbations and improve the prognosis. We also do not know whether their poor prognosis is necessarily mediated by the presence of exacerbations, or whether it is worsened by CBI by *P. aeruginosa* when people present few exacerbations, or none at all. An answer to this question could also open up possibilities of early treatment for people with CBI since at the moment some guidelines recommend that CBI should only be treated in the presence of exacerbations. Another point that has yet to be clarified is whether CBI by *P. aeruginosa* in people with COPD has the same clinical, prognostic, and therapeutic implications, regardless of any association with bronchiectasis.

With respect to asthma, we need to establish whether the concept of CBI is applicable here, as this would involve stronger symptoms and a poorer clinical evolution. If this was the case, it would, therefore, be necessary to define the patient phenotype, or degree of asthma severity, that would mark the threshold for conducting studies aimed at diagnosing CBI.

Furthermore, in the light of the growing threat of CBI by resistant bacteria, healthcare administration needs to take into account the possibility of transmission of *P. aeruginosa* by people with bronchiectasis and COPD in hospital settings [228], as has already occurred in CF [228,229]. The guidelines for both CF and bronchiectasis have formulated recommendations on how often sputum cultures should be performed and indications as to when invasive diagnostic explorations are appropriate [153,155]. Similar recommendations are needed for people with COPD and asthma, as well as an assessment of any need to modify their follow-up when their disease is associated with bronchiectasis. Moreover, it is necessary to find biomarkers that would allow us to predict which adults with CBI by *P. aeruginosa* will present a better or worse prognosis or a higher or lower number of exacerbations. Ideally, these biomarkers should also enable us to determine the effectiveness of treatment for exacerbations. Advances of this kind would allow us to introduce personalized management of adults with CBI.

Finally, further studies are required to determine whether the progressive development and standardization of techniques for identifying the pulmonary microbiome [230] can improve our management of people in clinical practice, especially in those cases where conventional cultures are insufficient for understanding why certain adults present an unfavorable evolution even though their management is theoretically correct. 

## Figures and Tables

**Table 1:** Examples of adaptative genes facilitating the survival of *P. aeruginosa* isolates (modified from Marvig-2015) [16].

Gene	Product/Function
*aceE*	Pyruvate dehydrogenase, quorum sensing
*algU*	Stress sigma factor regulating alginate production, biofilm formation
*ampC*	β-lactamase precursor, elongation, cell division
*exoS*	Effector protein, cytotoxic and antiphagocytic effects
*fim*	Fimbrial formation, motility, adhesion/attachment, receptor recognition
*ftsI*	Penicillin-binding protein, elongation, cell division
*fusA1*	Elongation factor G
*gyrA*	DNA gyrase subunit A, cell division
*gyrB*	DNA gyrase subunit B, cell division
*lasR*	Transcriptional regulator, quorum sensing
*mexA*	Multidrug efflux membrane fusion protein precursor
*mexB*	Multidrug efflux transporter
*mexS* *p*	Oxidoreductase involved in the regulation of multidrug efflux pump
*mexY*	Multidrug efflux transporter
*mexZ*	Transcriptional regulator of multidrug efflux pump
*muc*	Alginate production, biofilm formation
*nalD*	Transcriptional repressor of multidrug efflux pump
*oprD*	Outer membrane porin
*phz*	Phenazine synthesis, virulence factor regulator, quorum sensing
*pvdA*	Pioverdine, antibacterial effect, biofilm formation
*pelA*	Polysaccharide deacetylase
*pil*	Pili formation, motility, adhesion/attachment, receptor recognition
*rhl*	Elastase synthesis, quorum sensing
*rsml*	Rhamnolips synthesis, quorum sensing
*rbdA*	Protein with c-di-GMP cyclase and phosphodiesterase domain
*yecS*	L-cysteine transporter of ABC system

**Table 2:** Studies that have analyzed the various consequences of infection by *Pseudomonas aeruginosa* in people with cystic fibrosis.

Author/Year	Analysis	No.	Type of Study	Main Finding
Cuthbertson [45] 2020	Diversity of microbiome and lung function	Sputum samples from 299 people with CF in the USA and Europe	Transversal	The less the diversity in the microbiota, the worse the lung function. As lung function declines, recognized pathogens, particularly PA, predominate in CF. PA is associated with poorer lung function.
Avendaño-Ortiz [51] 2019	Immune response	32 people with CF, 19 with PA, 15 healthy	Case/control	Infection by PA gives rise to a reduced monocyte response to various stimuli.
Styleman [52] 2019	Lung function patterns	60 CF >16 years	Transversal	Infection by PA is associated with greater obstruction.
Acosta [44] 2018	Microbiota, factors associated with progression	Sputum sample from 104 people with CF aged from 18 to 22 years	Longitudinal	Reduced diversity and a greater presence of PA give rise to a more rapid decline in lung function and greater progression to severe forms leading to a transplant or death. No other microorganism increased the risk of progression.
Somayaji [53] 2017	Impact of PA Prairie Epidemic Strain (PES) on morbidity-mortality	274 adults with CF	Longitudinal 1980–2014	Infection by PES was associated with increased patient morbidity over three decades, manifested by a greater risk of respiratory death and/or lung transplant.
López-Causapé [48] 2017	Characterization of isolations of PA in Spain	24 CF units in Spain Sputum samples from 341 people with CF	Transversal	The PA isolations are very diverse and genetically unrelated in Spain, with multiple combinations of virulence factors and high levels of resistance to antimicrobial drugs (apart from colistin).
Zemanick [54] 2013	Consequences of primary infection by PA	838 people with CF <12 years, with no isolations of PA prior to inclusion	Longitudinal: EPIC, followed up from 2004 to 2006	The acquisition of PA was associated with a significant increase in the rates of exacerbations and crackles and wheezing.
De Dios-Caballero [47] 2016	Patterns of infection-colonization in people with CF in Spain	24 CF units in Spain Sputum samples from 341 people with CF	Transversal	Chronic bronchial infection by PA in 46% of people (29% in children and 63% in adults). Chronic bronchial infection by PA and methicillin-resistant *S. aureus* is associated with poorer lung function.
Mayer-Hamblett [55] 2014	Influence of PA phenotypes on severe exacerbations	649 children with CF primary PA infection 2594 isolations	Longitudinal: EPIC, 5.4 years of follow-up	Mucoid and altered motility phenotypes predicted the appearance of severe exacerbation.
Ramsey [56] 2014	Lung function, CT, inflammation by BAL	68 people with CF 48 healthy controls	Longitudinal 3 to 7 years Case/control	People with CF had poorer lung function. Bronchial infection by various microorganisms like PA was associated with poorer lung function.
Zemanick 2015 [57]	Relationship of microbiota, inflammation and lung function in exacerbations	21 people with CF. Sputum, FRT, and blood pre- and post- exacerbation	Transversal	Anaerobes identified in sputum via sequencing were associated with less inflammation and better lung function compared to PA in an early exacerbation.
Dill 2013 [58]	Quality of life and PA	333 adults with CF	Longitudinal	In the presence of *S. Aureus, Burkholderia* and PA were not predictors of any of the physical domains of quality of life.
Folescu [59] 2012	CT in people with PA	41 people with CF, 26 with chronic PA infection	Transversal, retrospective	The higher radiological scores in the PA group showed evidence of greater deterioration in lung function.
Mott [60] 2012	Progression in CT	143 people with CF from a screening program	Longitudinal 1 year	The radiological progression of bronchiectasis and air entrapment were associated with the severe CFTR genotype, worse neutrophilic inflammation and lung infection.
Ren [61] 2012	Lung function	93 pre-school children with CF	Longitudinal 2 years	Previous PA infection was associated with a higher rate of reduction in the FEF 25–75 z-score and mild thoracic-abdominal asynchrony in pre-school children with CF.
Taylor-Robinson [62] 2012	Decline in lung function	479 people with CF	Longitudinal 1969–2010	Infection by PA was associated with a significant increase in the rate of lung function decline (around 0.5%/year).
Sawicki [63] 2012	Treatment of chronic PA infection with inhaled tobramycin	12,740 people with CF	Longitudinal 6 years	Positive cultures for PA and *Burkholderia* were associated with increased mortality. The use of inhaled tobramycin reduced mortality.
Ashish [64] 2012	Impact of chronic infection by PA and LES-PA on quality of life (CFQ)	157 people with CF 43 with chronic PA, 93 with chronic LES-PA, and 20 with no PA	Transversal	People with LES-PA presented a poorer quality of life in most domains of the CFQ. People infected by PA only presented poorer scores than those not infected in the domain of bodily perception.
Konstan [65] 2012	Decline in lung function	4161 adults with CF	Longitudinal Follow-up of 3 to 25 years	The mean rates of reductions in the FEV1 were −1.92 from 18 to 24 years of age and −145 ≥ 25 years. In the 18–24-year-old group, *B. cepacia*, the use of pancreatic enzymes, multi-resistant PA, mucoid PA, and female gender predicted a greater decline in lung function.
Pillarisetti [66] 2011	Decline in lung function associated with inflammation and infection	37 lactating people with CF in screening program FRT/BAL	Longitudinal	The more neutrophil elastase in BAL, the poorer the lung function. Greater decline in lung function in people infected by *S. aureus* and PA.
Gangell [67] 2011	PA and inflammation	653 samples 215 people with CF aged up to 7 years	Prospective BAL	PA and *Aspergillus* were associated with higher levels of inflammation, particularly PA.
Rosenfeld [50] 2010	Factors associated with the early acquisition of PA in young children with CF	1117 children with CF but no PA (PA-Never) 583 children with CF and eradicated PA (PA-Past)	Longitudinal: EPIC, follow-up from 2004 to 2006	The PA-Never people had better lung function and fewer respiratory symptoms than those with a remote history of infection by PA.
Robinson [68] 2009	PA and CT	25 children with mild–moderate CF CT, FRT, cultures	Transversal	The CT scores had a high correlation with the acquisition of PA, which is a clinically significant measure of the progression of lung disease.
Konstan [69] 2007	Decline in lung function	4866 children and adolescents with CF	Longitudinal Follow-up of 3 to 6 years	Colonization by PA was associated with an increase in the rate of FEV1 loss (0.31% per year in the group aged 6–8 years and 0.22% in the 9–12 group).
Courtney [70] 2007	Microbiology, genetics, and lung function	183 adults with CF	Longitudinal 1995–2005	People infected with PA and *Burkholderia* presented higher mortality, and these pathogens were the main predictors of mortality.
Taccetti [71] 2005	Early eradication of PA	Ciprofloxacin plus colistin to eradicate PA in 47 adults with CF	Case/control	After early antibiotic therapy: Period free of PA of 18 (4–80) months. Delayed deterioration of lung function in comparison with people with chronic infection. Prevention of appearance of strains of PA resistant to antibiotics. New acquisition with different genotypes of PA in 73%.
Li [72] 2005	Relationship of mucous PA with morbidity, symptoms, antibodies, X-R and FRT	56 adults with CF	Longitudinal from birth to 16 years (1985 to 2004)	The transition time of non-mucoid PA was relatively long (mean, 10.9 years). The deterioration in cough scores and X-R and the decline in lung function correlated with the transition from non-mucoid to mucoid PA.
Coburn [43] 2015	Microbiome: Sequencing of ribosomal RNA	Sputum samples from 269 people with CF	Transversal	Greater diversity in the microbiome at a lower age. Less diversity correlated with poorer lung function. Greater prevalence and relative abundance of PA and *Burkholderia* in older people. PA was associated with poorer lung function.
West [27] 2002	Cultures and antibodies against PA	68 children screened for CF	Longitudinal 1985–2000	In CF screening, the antibodies against PA were present 6 to 12 months before any isolation in respiratory secretions.
Nixon [33] 2001	Relationship between inflammation and infection with symptoms and lung function	CF children aged under 3 years BAL Lung function	Neonatal screening	Infection of the lower airways by various microorganisms as demonstrated by BAL was associated with a 10% reduction in the FEV (0.5) compared to subjects with no infection.
Emerson [49] 2002	PA as a prognostic factor for morbidity and mortality	3323 people with CF from the CFF-PR	Longitudinal 1990–1998	People with positive cultures for PA had a 2.6 times higher risk of death at 8 years and they presented a lower percentage of FEV1 and weight percentile, as well as a greater risk of hospitalization and respiratory exacerbation in the follow-up.
Nixon [33] 2001	Impact of early primary infection by PA. BAL	56 people with CF screened from 1990 to 1992	Longitudinal Follow-up of up to 7 years	Four children infected with mucoid, multi-resistant PA died under the age of 7. Infection by PA was associated with more morbidity, greater lung function decline, longer hospitalizations, and higher mortality.
Navarro [73] 2001	Lung function	European CF Registry. 7010 people with CF	Follow-up of 6 years	PA was associated with lower FEV1.
Kosorok [34] 2001	Impact of PA Radiological score FEV1	56 children from CF screening program	Longitudinal	The rate of lowering of the radiological score increased after the acquisition of PA (from 0.45 to 1.40 points/year; *p* < 0.001). Lung function decline speeded up after the acquisition of PA, (previous FEV1/FVC decline of 1.29%/year; subsequently it was 1.81%; *p* = 0.001).
Hudson [74] 1993	Early infection by PA and prognosis	81 children with CF aged under 2 years at the start	Longitudinal Follow-up of 5.4 to 13 years	Initial PA at 2 years or under was associated with increased morbidity (lower radiological score and poorer lung function). Detection of PA and *S. aureus* in cultures was associated with a higher mortality rate in the first 10 years after the diagnosis.
Henry [75] 1992	Impact on survival of mucoid isolates of PA	81 children with CF Sputum samples Lung function	Longitudinal 8 years or mortality	FEV1 was the main prognostic factor for mortality. The identification of mucoid forms of PA was an unfavorable prognostic factor.
Pamucku [76] 1995	Infection by PA and lung function	192 children with CF	Longitudinal 1982–1992	FEV1 loss was more significant in people colonized by PA than those who were not.
Kerem [77] 1990	Infection by PA and evolution of la lung function	895 children with CF	Longitudinal from 1975 to 1998	Prevalence of PA of 82%. At the age of 7 years, the mean percentage of predicted FEV1 was 10% lower in those children already colonized by PA, compared to those who were not (*p* < 0.01).

PA: *Pseudomonas aeruginosa*; LES: Liverpool Epidemic Strain; CT: computerized tomography; X-R: chest X-ray; CFTR: cystic fibrosis transmembrane conductance regulator of cystic fibrosis; CFQ: quality of life questionnaire for cystic fibrosis; BAL: bronchoalveolar lavage; FRT: functional respiratory tests; FEV1: forced expiratory volume in 1 s; FEF: forced expiratory flow; EPIC: Early Pseudomonas Infection Control Observational Study. Prospective cohort study analyzing risk factors and clinical results associated with the early acquisition of PA in children with cystic fibrosis; CFF-PR: Cystic Fibrosis Foundation Patient Registry.

**Table 3:** Main studies showing the clinical impact of *Pseudomonas aeruginosa* on people with bronchiectasis.

Authors Date	Study Design	Site/Year of Study	Mean Age in Years	People Included	% of Colonization by *P. aeruginosa*	Years of Follow-Up	Results
Hernandez 2002 [143]	Prospective Cohort	Spain 1999–2000	56	70	20	Transversal	QoL, FEV1, FVC
Martinez-García 2007 [132]	Prospective	Spain 2003	69.9	76	19.7	2 years	FEV1 loss
Loebinger 2009 [138]	Longitudinal	UK 1994–2009	51.7	91	22	13	Mortality
Chalmers 2014 [144]	Prospective Cohort	UK					Pulmonary exacerbations, FEV1, FVC, QoL, radiographical severity, hospitalizations, mortality
		2008–12	67	608	11.5	4	
		2011–14	68	289	14	3	
Martinez-García 2014 [131]	Retrospective Multi-center	Spain Prior to 2005	58.7	819	31.8	5	Mortality
Goeminne 2014 [145]	Prospective Cohort	Belgium 2006–12	68	253	7.9	5.18	Pulmonary exacerbations, FEV1, FVC, radiographical severity, hospitalizations, mortality
Mc Donnell 2015 [146]	Retrospective	UK 2007–9	63	212	16	5	Pulmonary exacerbations, hospitalization, FEV1, FVC, radiographical severity, mortality
Finch 2015 [4]	Systematic review of 21 studies	Italy, UK, Belgium, Spain, Australia, Ireland, China 1990–2014	51.7–68	3683	21.4	Transversal 13 years	Pulmonary exacerbations, FEV1, FVC, QoL, radiographical severity, hospitalization, mortality
Aliberti 2016 [147]	Prospective Cohort	5 European countries	60	1145	16	3	Clusters, pulmonary exacerbations, QoL, mortality
De La Rosa 2018 [148]	Retrospective Multi-center	Spain	67	456	37.2		Costs
Araujo 2017 [149]	Prospective Multi-center	Europe Israel	67	2596	15	5	Mortality Pulmonary exacerbations QoL
Martínez-Garcia 2020 [133]	Prospective RIBRON	Spain	69.1	849	25.7	1–4	FEV1 loss

FEV1: forced expiratory volume in first second; FVC: forced vital capacity; QoL: quality of life.
